# Targeting HER2 expression in cancer: New drugs and new indications

**DOI:** 10.17305/bjbms.2020.4908

**Published:** 2021-02

**Authors:** Semir Vranić, Semir Bešlija, Zoran Gatalica

**Affiliations:** 1 College of Medicine, QU Health, Qatar University, Doha, Qatar; 2 Department of Oncology, Clinical Center of the University of Sarajevo, Sarajevo, Bosnia and Herzegovina; 3 Creighton University School of Medicine, Phoenix, Arizona, United States; 4 Oklahoma University College of Medicine, Oklahoma City, Oklahoma, United States

**Keywords:** HER2, targeted therapy, mutations, amplification

## Abstract

Functional activation of human epidermal growth factor receptor 2 (HER2) has been shown to strongly promote carcinogenesis, leading to the investigation of HER2-directed agents in cancers with *HER2* genomic alterations. This has been best documented in the context of *HER2* gene amplification in breast and gastric/gastroesophageal junction carcinomas for which several HER2-directed agents are available and have become a part of standard treatment regimens. Somatic *HER2* gene mutations have been recently described at low frequency in a variety of human cancers and have emerged as a novel predictive biomarker for HER2-directed therapies. Preclinical data also indicate that activating *HER2* mutations are potent oncogenic drivers in a manner that is analogous to *HER2* amplification. *HER2* mutations may clinically confer sensitivity to HER2-directed agents as recently shown in a phase II clinical trial with antibody-drug conjugate against HER2 trastuzumab deruxtecan in patients with non-squamous non-small cell lung carcinoma.

The oncogenic potential and activation of human­ ­epidermal growth factor receptor 2 (HER2) has been well established in several human malignancies, most notably in breast and gastric/gastroesophageal junction (GEJ) carcinomas. The primary mechanism of the HER2 activation in these cancers is *HER2* gene amplification that leads to the complete HER2 protein overexpression on the cellular membrane [1,2]. In recent years, other genomic alterations of *HER2* have also been recognized leading to protein activation among which *HER2* gene mutations represent the most important form [3]. *HER2* mutations are usually of activating type and the majority of them are seen without concurrent *HER2* gene amplification [3-5]. The highest prevalence (>10%) of *HER2* mutations has been observed in prostate neuroendocrine carcinomas, metastatic cutaneous squamous cell carcinomas and urothelial bladder carcinoma. Additionally, *HER2* mutations have also been reported in common cancers such as pulmonary, colorectal and breast cancers, indicating a potential for HER2-directed therapies in these cancers [4,6-12]. *HER2* mutations are enriched in certain specific histological subtypes, for instance in invasive lobular carcinoma of the breast (5-18%) [13-15] and in ~2-3% of pulmonary adenocarcinomas [16-21]. Recently, *HER2* mutations were reported to occur more frequently in microsatellite instable (MSI-H) colorectal carcinomas than in microsatellite stable (MSS) cases [10, 11]. *HER2* genomic alterations were also enriched in RAS wild-type and anti-EGFR therapy resistant colorectal carcinomas [22]. Studies in non-small cell lung carcinomas (NSCLC) revealed mutations affecting predominantly exon 20 and were seen without amplification of *HER2* gene. In addition, *HER2* mutations were mutually exclusive with other common oncogenic drivers in NSCLC. In contrast to epidermal growth factor receptor (*EGFR)* mutations, the frequency of *HER2* mutations appear to be similar between Asian and Caucasian populations [16].

The anti-HER2 antibody trastuzumab has been a cornerstone and effective therapy in treatment of HER2-positive breast and gastric cancers. However, the number of approved anti-HER2 therapeutic agents has been markedly expanded in recent years, with the addition of tyrosine kinase inhibitors (lapatinib, neratinib, tucatinib), antibodies (pertuzumab), and antibody-drug conjugates [ado-trastuzumab emtansine (T-DM1)] and trastuzumab deruxtecan (DS-8201)] (summarized in Table 1) [3]. Used alone or in combination with other targeting agents or conventional chemotherapeutics, these anti-HER2 agents have remarkably improved the outcome of patients with HER2-positive breast cancer [23, 24].

**Table 1:**
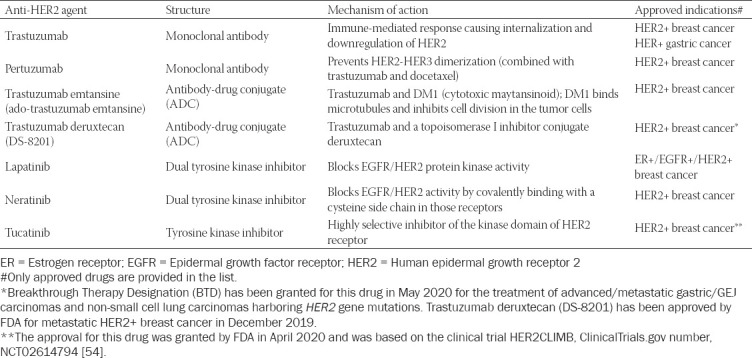
Overview of the anti-HER2 agents that have been approved in breast and gastric cancers

One of the most recent antibody-drug conjugates (ADC) against HER2 is trastuzumab deruxtecan (Enhertu; DS-8201; AstraZeneca and Daiichi Sankyo Company, Limited (Daiichi Sankyo). It is composed of an anti-HER2 antibody (trastuzumab), a cleavable tetrapeptide-based linker, and a cytotoxic topoisomerase I (TOP1) inhibitor (“payload”) (Table 1). Previously, it was published that TOP1 was overexpressed in 63% of all invasive breast carcinomas [25]. Enhertu appears to exhibit a higher drug-to-antibody ratio than another anti-HER2 ADC trastuzumab emtansine (Kadsyla) (approximately 8 vs. 3-4) while retaining a favorable pharmacokinetic characteristics [26]. In contrast to trastuzumab emtansine, trastuzumab deruxtecan has a released payload that may easily cross the cell membrane, which potentially allows for potent cytotoxic effects on cancer cells regardless of target expression [27-29]. In addition, the released payload has a substantially shorter half-life, which may minimize systemic exposure and potential side effects [30]. In breast cancer, trastuzumab deruxtecan has been shown to exhibit a durable therapeutic activity in a population of heavily pretreated patients (≥2 prior anti–HER2-based regimens) with advanced HER2-positive breast cancer [31]. This phase 2 study (II DESTINY-Breast01 trial) revealed that a response to trastuzumab deruxtecan was achieved in 60.9% of the patients of which 6% had a complete response (CR) while 54.9% of patients had a partial response [31]. The preliminary data from the II DESTINY-Breast01 trial were presented at the San Antonio 2019 meeting [32] and led to the accelerated approval by the Food and Drug Administration (FDA) in December 2019.

The results from another ongoing trial named Phase II DESTINY-Lung01 trial have just been presented at the 2020 American Society of Clinical Oncology (ASCO) Virtual Scientific Program [33]. This trial showed that trastuzumab deruxtecan also achieved a clinically significant tumor response in patients with HER2-mutant advanced non-squamous NSCLC whose disease had progressed following one or more previous systemic therapies (chemotherapy or immunotherapy with immune checkpoint inhibitors against PD-1/PD-L1) [33]. The confirmed objective response rate (ORR) was achieved in 61.9% of patients treated with trastuzumab deruxtecan monotherapy (6.4mg/kg). A disease control rate (DCR) of 90.5% with a median progression-free survival (PFS) of 14 months were remarkable achievements in this study. However, other relevant endpoints such as a median duration of response and overall survival (OS) have not been reached at the time of data cut-off [33]. Nevertheless, these encouraging results, after many failed trials and extensive research on HER2 in NSCLC [34-36], may pave a new way for the treatment of a small subset of NSCLC harboring *HER2* gene mutations. Interestingly, for the predictive purposes HER2-overexpressing non-squamous NSCLC (defined as scores 2+ and 3+ by immunohistochemistry/IHC/) or non-squamous NSCLC harboring a HER2-activating mutation, were used in this trial. From the provided abstract [33], it is not clear whether the differences in a treatment response were observed between HER2-mutant NSCLC and HER2-overexpressing NSCLC (2+ and 3+ scores by IHC). In contrast, an ongoing, phase II trial with trastuzumab deruxtecan in advanced gastric/GEJ carcinomas (DESTINY-Gastric01) enrolled only those patients whose cancers were HER2-positive (Score 3+ by IHC or 2+ IHC with confirmatory *HER2* amplification by in situ hybridization assay). A clinical trial on NSCLC with ADC trastuzumab emtansine (T-DM1) reported responses to the targeted drug only in IHC 3+ NSCLC while no responses were found in 2+ NSCLC cases [37]. It is well-known that HER2 status assessed by IHC (particularly score 2+) is not an optimal approach for selection of the patients having a mutant *HER2* cancer as recently shown in case of colorectal carcinoma [38]. Apart from the well-documented examples of mutation-specific IHC antibodies (e.g. BRAF p.V600E, IDH1 p.R132H, or H3K27M), which tend to correlate well with the specific mutations [39], IHC antibodies that target specific proteins may lack correlation with the DNA-level mutational events. There are several possible reasons including the fact that the mutation may not result in (increased) protein expression (e.g. due to transcriptomic silencing of genetic variant or discordance between the DNA alterations and RNA expression)[40], hence IHC may be consequently negative/low positive; another possibility is that the antibody may target a specific epitope that may or may not be altered by the mutation. In case of trastuzumab deruxtecan, another important therapeutic target is TOP1, which is inhibited by the ADC payload deruxtecan. TOP1 is an enzyme with an active role in DNA function by the cleavage one of the two backbones in double-stranded DNA enabling the double helix to be untwisted [41]. TOP1 status has been extensively studied by immunohistochemistry, most notably in colorectal carcinoma where it has been shown to predict a response to irinotecan-based chemotherapy [42,43]. It is well-known that irinotecan may reversibly stabilize the TOP1 cleavable complex, resulting in single-strand DNA breaks and ultimate cell death [44]. In addition, a high TOP1 expression has also been demonstrated in several other cancers, including small cell lung, gastric/gastroesophageal, esophageal, thymic, anal, breast, prostate and poorly differentiated neuroendocrine carcinomas [25]. The same study as well as several other studies also revealed a common TOP1 overexpression in NSCLC [45,46]. Another ADC sacituzumab govitecan-hziy (TRODELVY, Immunomedics, Inc.) was recently approved for the patients with metastatic triple-negative breast cancer (TNBC). It represents an anti-Trop-2 (=trophoblast cell-surface antigen 2) ADC and contains irinotecan metabolite, SN-38 that is conjugated to a humanized anti-TROP-2 antibody (sacituzumab govitecan) [47]. Although both Trop-2 and TOP1 expression have been well-documented in various cancers including breast cancer [25,48-52], predictive testing was not conducted in this trial (IMMU-132-01 (NCT 01631552) clinical trial [53].

In short, recent data indicate that *HER2* mutations may be successfully targeted with the available anti-HER2 treatment modalities. ADC such as trastuzumab deruxtecan represent novel promising therapeutic means for the patients with advanced non-squamous NSCLC. Despite the remarkable achievements, we believe that further efforts should be made to optimize the treatment with these ADC where molecular targets are well characterized and may be easily assessed prior to targeted treatments.
